# Supplementary figures and images for: A hybrid epithelial-mesenchymal transition program enables basal epithelial cells to bypass stress-induced stasis and contributes to a metaplastic breast cancer progenitor state
Source: Breast Cancer Res. 2024 Dec 18;26:184. doi: 10.1186/s13058-024-01920-8 (PMC11657373; doi:10.1186/s13058-024-01920-8)

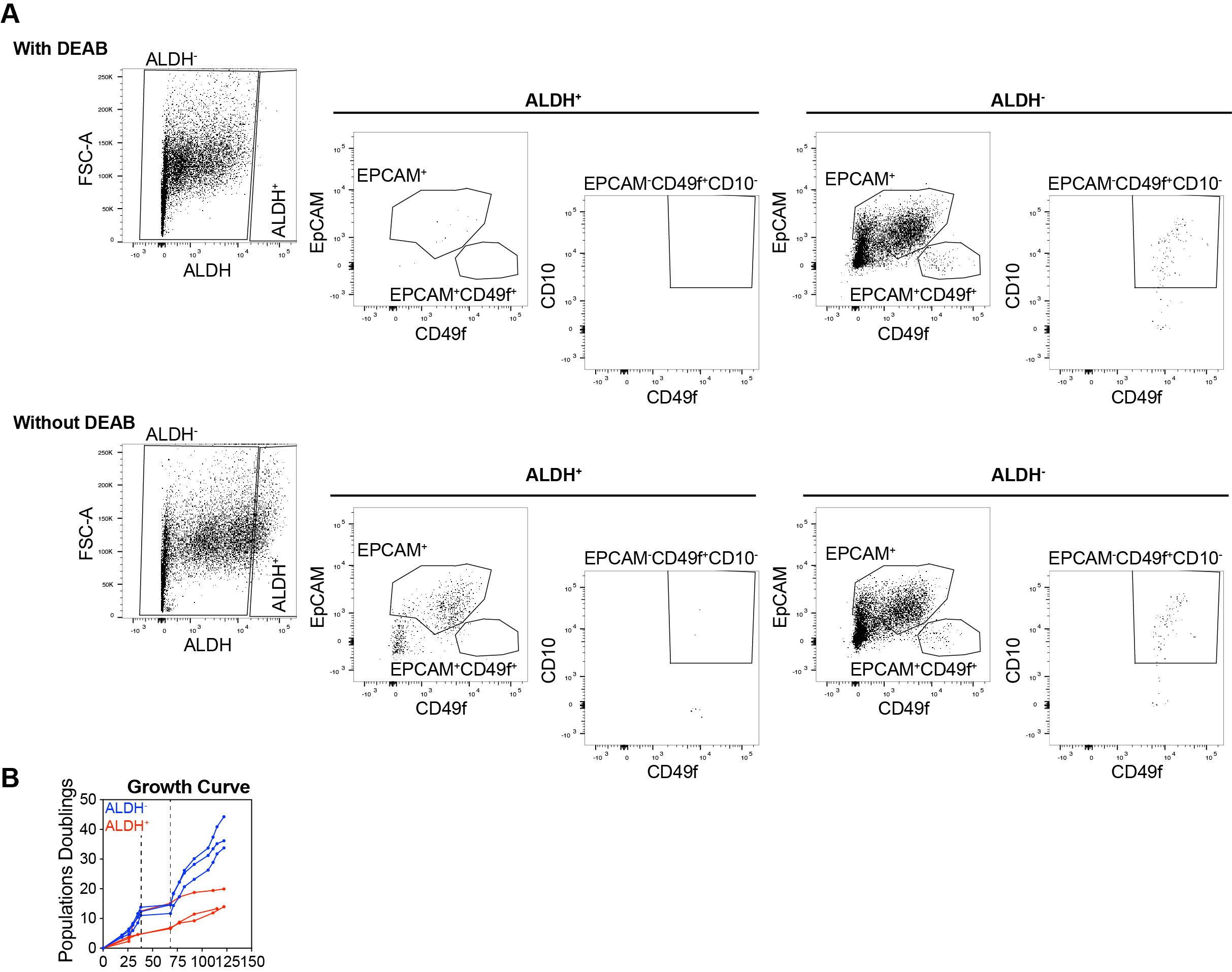

Supplement: Supplementary file 3 — Supplementary Material 3: Fig. S1 Rare variants were not derived from ALDH+ cells. A Flow cytometry gating strategy for identifying ALDEFLUOR-positive cells. DEAB was used to establish the gating for ALDEFLUOR positivity in cells that were live (DAPI-) and lineage-negative (exclusion of CD2+, CD3+, CD4+, CD16+, CD64+, CD31+, and/or CD45+ cells). ALDEFLUOR-positive and -negative cells were visualized based on their expression of EPCAM (PerCP-Cy5.5) and CD49f (APC) expression. Basal cells (EpCAM-CD49f+) were further distinguished based on their expression of CD10 (PE). B ALDH+ and ALDH- epithelial cells were sorted out and cultured separately in MEGM. cells were counted at each passage, and the growth curve of the cumulative population doubling over time was plotted. [file 13058_2024_1920_MOESM3_ESM.jpg]

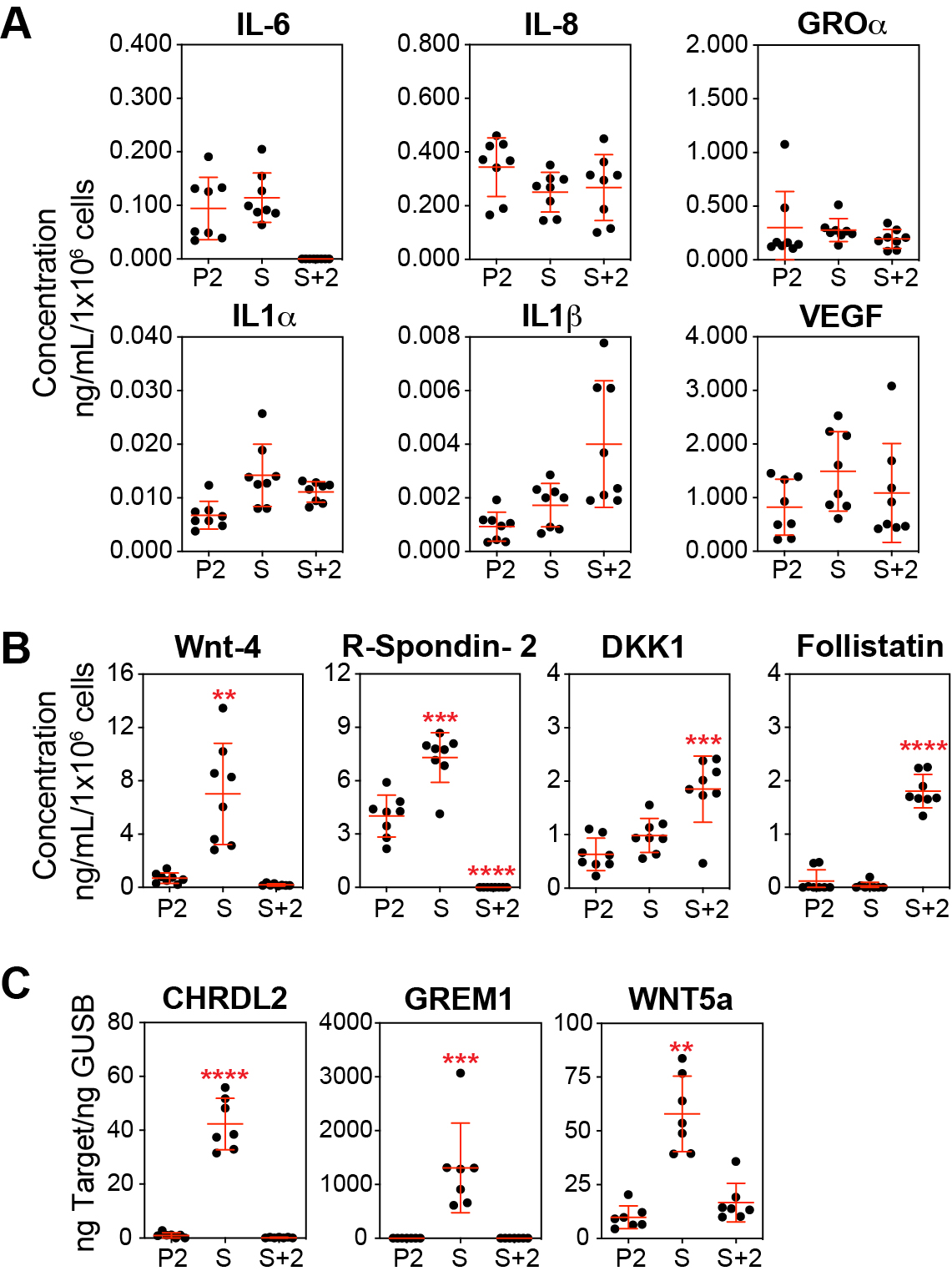

Supplement: Supplementary file 4 — Supplementary Material 4: Fig. S2 Secretome analysis of HMEC. A Sandwich ELISA kits were used to determine the concentrations of IL-6, IL-8, GRO, IL-1α, IL-1β, and VEGF based on standard curves. Equal numbers of cells were harvested from P2, S, and S+2 cultures and cultured in basal medium (without supplements) for 24 h. Concentrations were normalized to the final cell number. B Same as A, for Wnt-4, R-Spondin-2, DKK1, and Follistatin. C For CHRDL2, GREM1, and WNT5A, for which sandwich ELISA kits were unavailable, expression was analyzed by qPCR using cDNA produced from P2, S, and S+2 cells. [file 13058_2024_1920_MOESM4_ESM.jpg]

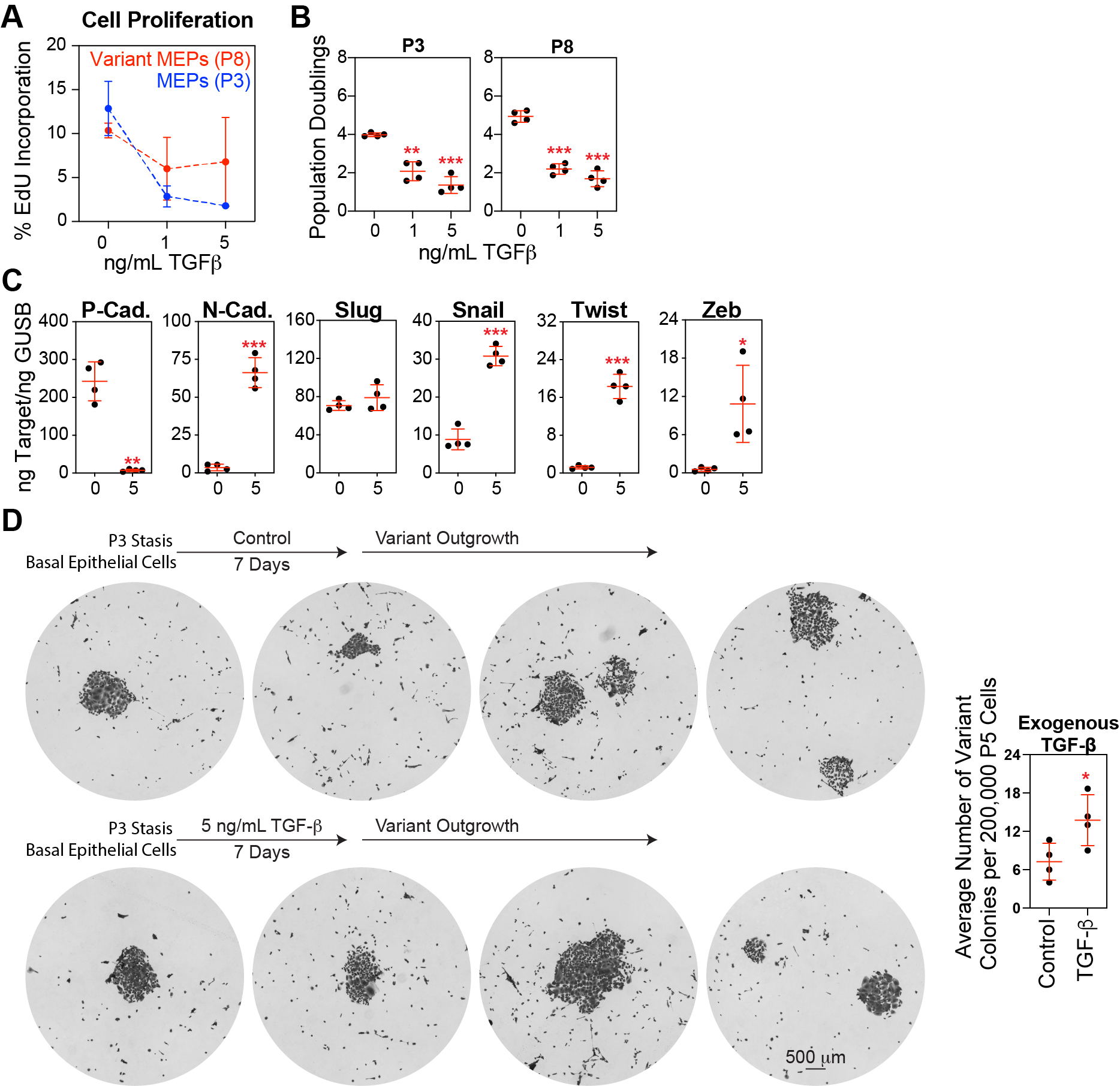

Supplement: Supplementary file 5 — Supplementary Material 5: Fig. S3 Exogenous TGF-β produces growth arrest in both the P3 and P8 (variant) basal epithelial cells. A Cell proliferation was measured in P3 and P8 cultures treated with 0, 1, or 5 ng/mL TGF-β for 48 h and pulsed with 10 µM EdU for 1 h using the Click-iT EdU cell proliferation assay. B Total populations doubling were calculated for P3 and P8 cultures treated with 0, 1, or 5 ng/mL TGF-β for seven days. C P5 stasis cultures were separated into six-well plates and treated with 0, 1, or 5 ng/mL TGF-β for seven days followed by 7 days of recovery in fresh MEGM. The number of variant colonies was quantified following staining with crystal violet. [file 13058_2024_1920_MOESM5_ESM.jpg]

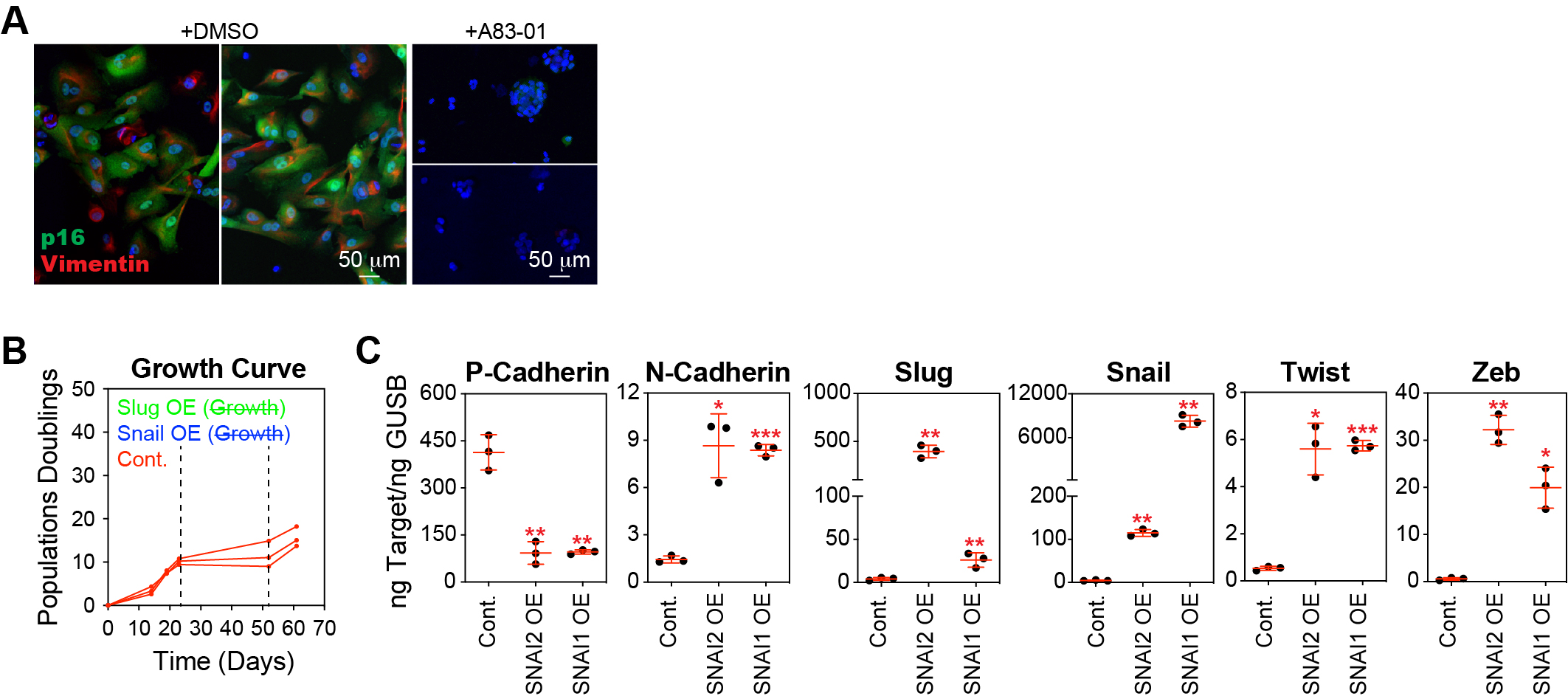

Supplement: Supplementary file 6 — Supplementary Material 6: Fig. S4 Growth arrest induced by snail or slug overexpression. A Representative images of P3 basal epithelial cells treated with 500 nM A83-01 or DMSO, immunostained with antibodies against p16 and vimentin and counterstained with DAPI. B Basal epithelial cells (P1) were transduced with lentiviral constructs containing Slug, Snail or GFP (control). After selection (1 μg/mL puromycin), Snail overexpressing (OE), Slug OE, and control cells were expanded in MEGM medium. At each passage, cells were counted to produce a growth curve of cumulative population doubling over time. Only control cells could be passaged; Snail OE and Slug OE were completely growth-arrested following selection. A second set of transduced basal epithelial cells was generated and collected immediately after selection. C Expression of P-cadherin, N-cadherin, Slug, Snail, Twist, and Zeb and was analyzed by qPCR using cDNA produced from basal epithelial cells transduced with Snail OE, Slug OE, or control vectors. [file 13058_2024_1920_MOESM6_ESM.jpg]

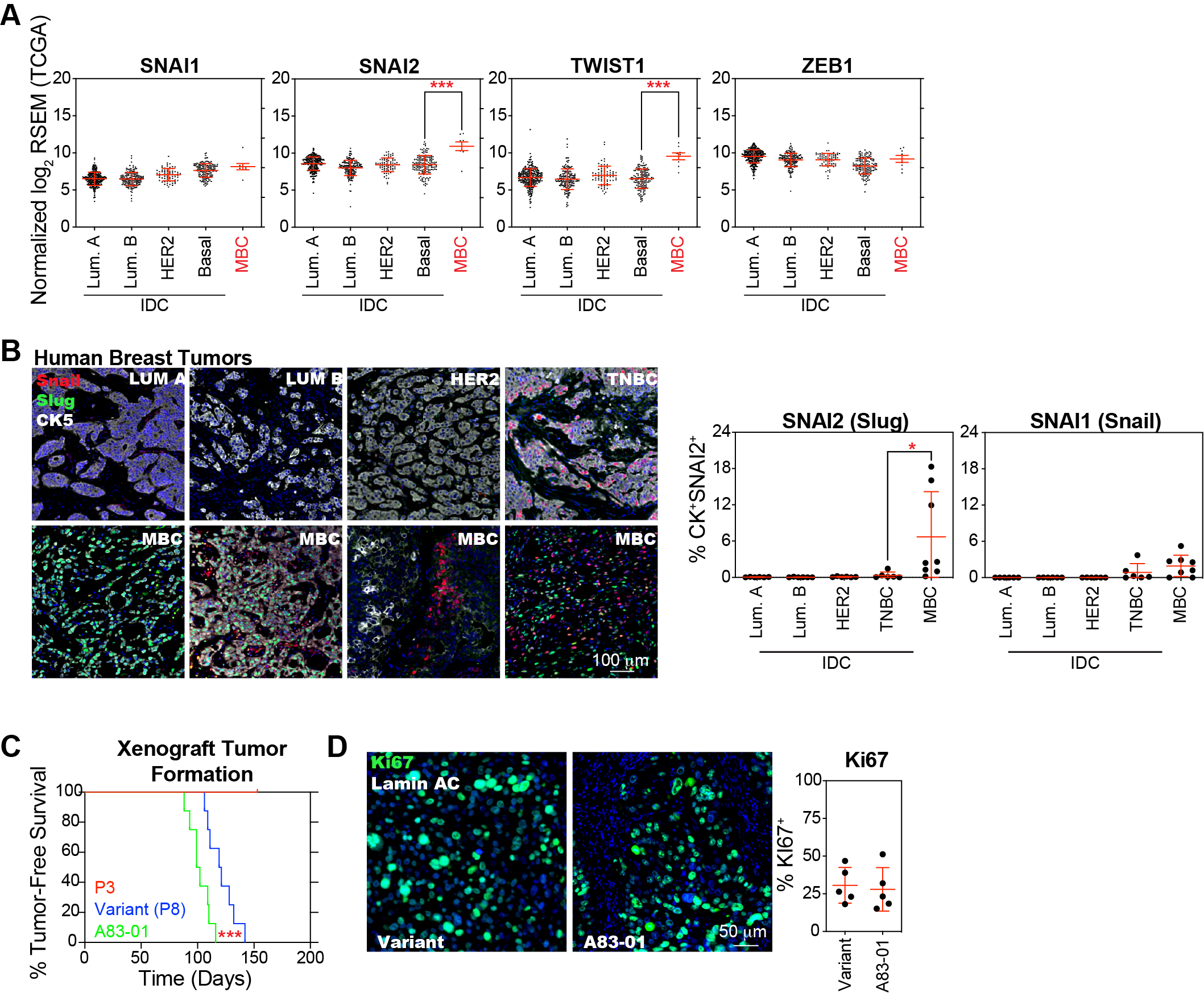

Supplement: Supplementary file 7 — Supplementary Material 7: Fig. S5 Higher levels of slug were characteristic of MBCs. A Using TCGA data accessed through https://www.cbioportal.org, we analyzed the expression levels of Snail (SNAI1), Slug (SNAI2), Twist (TWIST1), and Zeb (ZEB1) in bulk RNA-sequencing data from eight metaplastic breast cancer cases compared to invasive ductal carcinoma cases separated into major intrinsic subtypes. B FFPE sections from 24 cases of invasive ductal carcinoma (six from each subtype) and eight cases of metaplastic breast cancer were subjected to multiplexed immunohistochemical analysis for Snail, Slug, and cytokeratin 5 (CK5). Quantification was performed using QuPath software, and cells were segmented and classified based on intensity thresholds determined from unstained control slides for each individual marker. C Kaplan-Meier analysis of P3 basal epithelial cells (red), variant basal epithelial cells (blue), and A83-01-treated basal epithelial cells (green). The x-axis represents time (days), and the y-axis represents the proportion of tumor-free mice. Eight mice per group, Statistical analysis of tumor formation rates between the variant and A83-01 groups was performed using the log-rank test for trend (D) Variants and A83-01-treated oncogenically transformed basal epithelial cells (4 × 10^6^) were injected subcutaneously with 50% Matrigel into 8-to 12-week-old female NSG mice. Tumors formed over a period of 3–6 months. A83-01 was not administered to mice. FFPE sections from five tumors were subjected to multiplexed immunohistochemical analysis for Ki67. Human-specific lamin A/C was used to identify the tumor cells. Quantification was performed using QuPath software, and cells were segmented and classified based on intensity thresholds determined from unstained control slides for each individual marker. [file 13058_2024_1920_MOESM7_ESM.jpg]

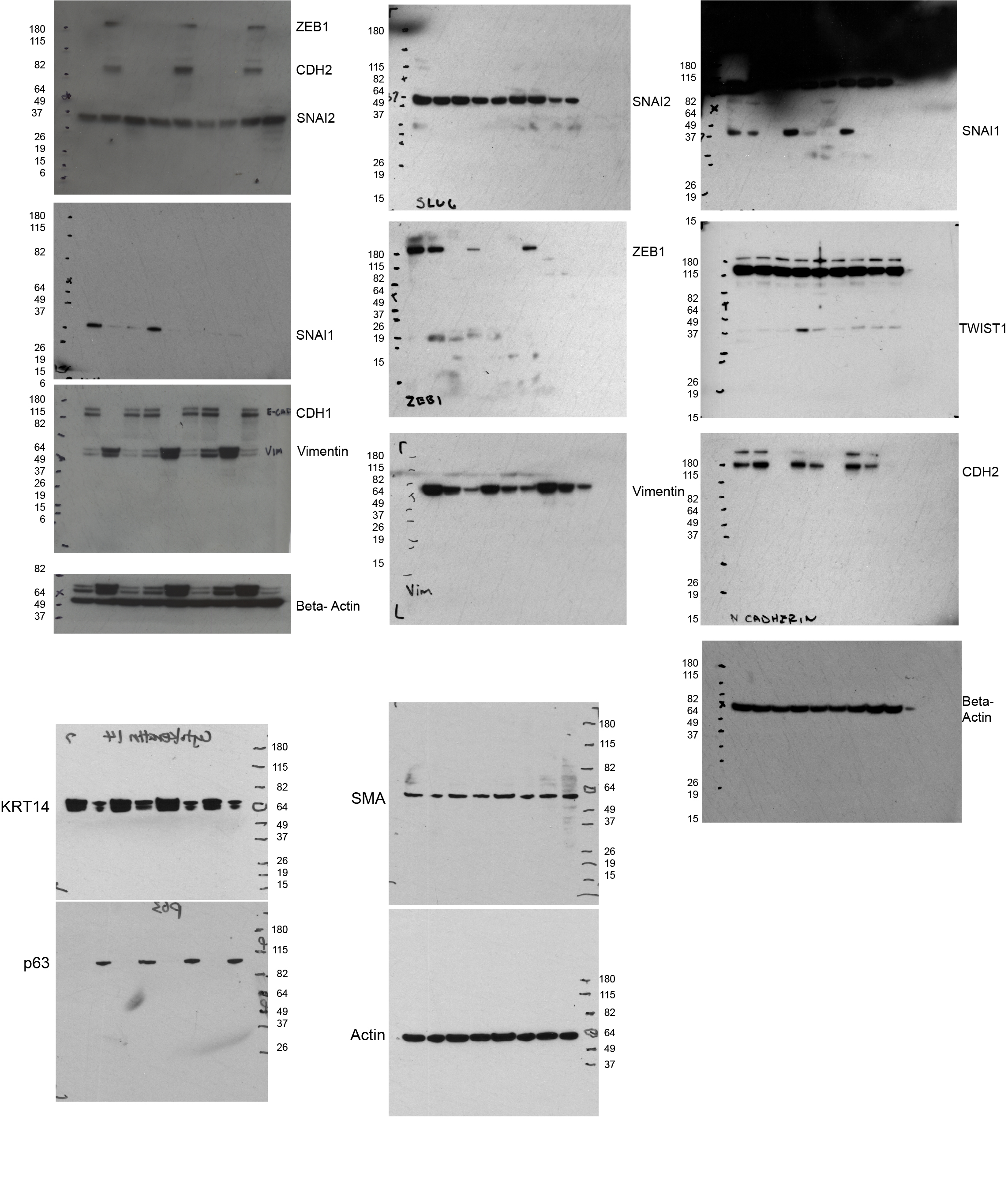

Supplement: Supplementary file 8 — Supplementary Material 8: Fig. S6 Full western blots. Western blots were developed using X-ray Film to visualize the chemiluminescence signal. [file 13058_2024_1920_MOESM8_ESM.jpg]

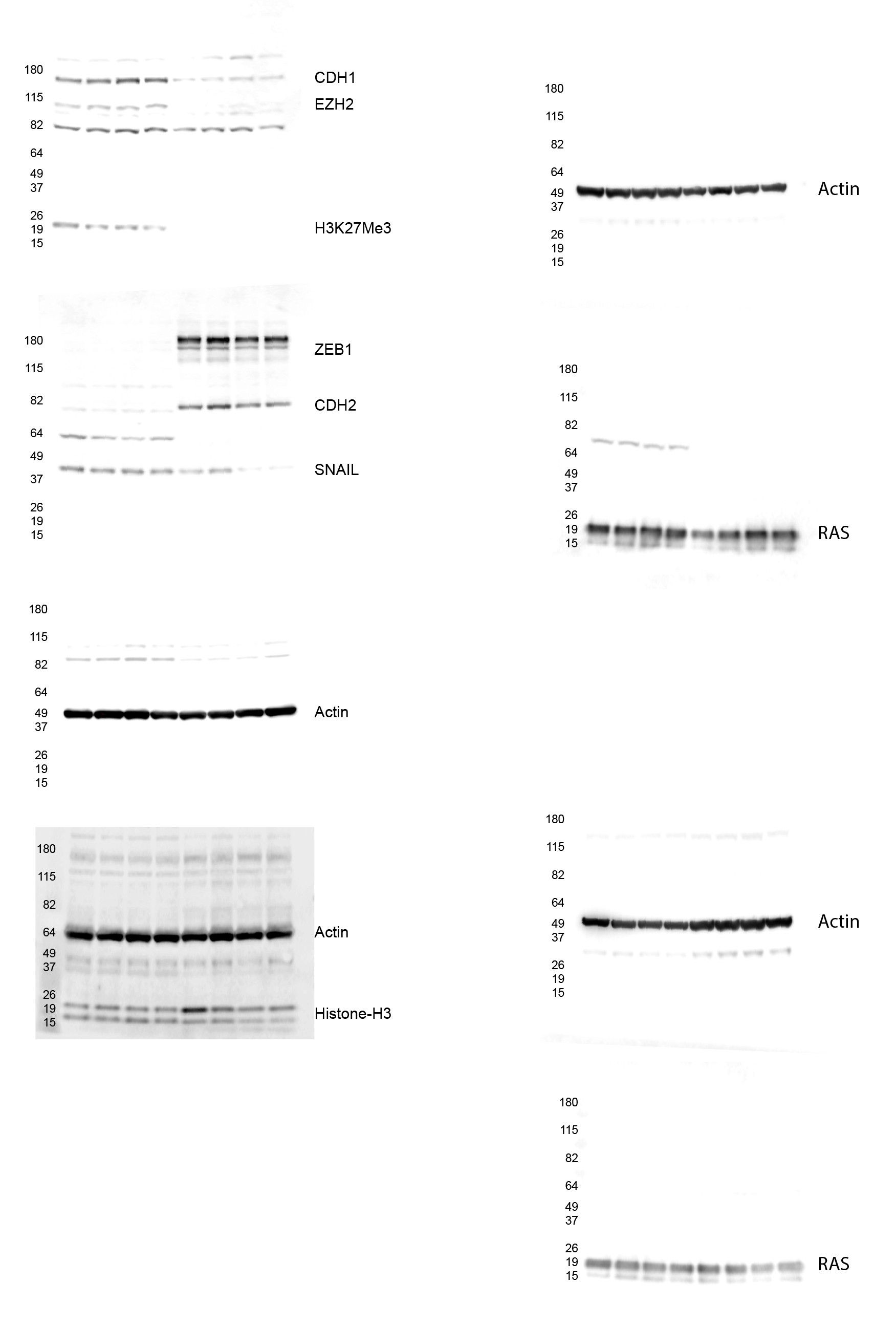

Supplement: Supplementary file 9 — Supplementary Material 9: Fig. S7 Full western blots. Western blots were developed using digital imaging to visualize the chemiluminescence signal. [file 13058_2024_1920_MOESM9_ESM.jpg]
